# Supplementary material for: Eccentric force and electromyogram comparison between the eccentric phase of the Nordic hamstring exercise and the razor hamstring curl
Source: PLoS One. 2023 Dec 15;18(12):e0293938. doi: 10.1371/journal.pone.0293938 (PMC10723670; doi:10.1371/journal.pone.0293938)
Supplement: Attachment — Submitted filename: Response to Reviewers.docx [file pone.0293938.s003.docx]

Thank you very much for reviewing our manuscript and providing valuable comments and suggestions that have helped us improve our manuscript. We have responded to your comments below and revised our manuscript accordingly.

In the revised manuscript, the changes are highlighted in yellow with red letters.

We hope that you will find our responses and revisions, considering your comments, adequate and that the manuscript has improved. We appreciate your further review of our manuscript and hope we have an opportunity to publish it in this journal.

Reviewer #1

1. In my opinion, the sample size is low to have robust results. It would be interesting to add more data to the study. As for the rest of the comments, the authors have reviewed them satisfactorily.

**Response**: Thank you very much for your indication. We have mentioned this in the limitation, but as you pointed out, we are considering the possibility that the sample size is small. Therefore, we believe it is necessary to conduct large-scale studies with larger sample sizes in the future.

Reviewer #4

1. Please rewrite L88-L91. The conjunction "therefore" has been followed. Please reconsider the connection of these sentences.

**Response**: Thank you very much for your indication. The conjunction "therefore" was used in succession, which made the sentences less connected. We have revised the text to take this into account and would appreciate it if you could confirm this.

**Change:** lines 86-89

Measuring peak eccentric force in addition to s-EMG can provide insights into the load on each muscle during NHE and RHC. However, no previous studies have compared peak eccentric force during NHE and RHC.
